# Effect of Mir-4270 Inhibitor and Mimic on Viability and Stemness in Gastric Cancer Stem-Like Cells Derived from MKN-45 Cell Line

**DOI:** 10.52547/ibj.3851

**Published:** 2023-01-23

**Authors:** Hassan Akrami, Seyedeh Azra Shamsdin, Yousef Nikmanesh, Mohammadreza Fattahi

**Affiliations:** Gastroenterohepatology Research Center, Shiraz University of Medical Sciences, Shiraz, Iran

**Keywords:** MicroRNAs, Neoplastic stem cells, Side-population cells

## Abstract

**Background:**

MiRNAs are significant regulatory factors in stem cell proliferation, and alteration in miRNA expression influences the viability and gene expression of cancer stem cells. Herein, we evaluated the effect of the hsa-miR-4270 inhibitor and mimic on the expression of stem cell markers in GCSCs.

**Methods:**

GCSCs were isolated from the MKN-45 cell line by a non-adherent surface system. The cells were confirmed by differentiation assays using dexamethasone and insulin as adipogenesis-inducing agents and also staurosporine as a neural-inducing agent. Isolated GCSCs were treated with different concentrations (0, 15, 20, 25, 30, 40, 50, and 60 nM) of hsa-miR-4270 inhibitor and mimic. The cell viability was determined by Trypan blue method. Transcription of the stem cell marker genes, including *CD44*, *OCT3/4*, *SOX2*, *Nanog*, and *KLF4*, was evaluated by quantitative real-time RT-PCR.

**Results:**

The results showed that GCSCs were differentiated into adipose cells using dexamethasone and insulin, and neural cells by staurosporine. Treatment of the GCSCs with hsa-miR-4270 inhibitor decreased the cell viability and downregulated *OCT3/4*, *CD44*, and *Nanog* genes to 86%, 79%, and 91% respectively. Also, *SOX2* and *KLF4 *were overexpressed to 8.1- and 1.94-folds, respectively. However, hsa-miR-4270 mimic had opposite effects on the cell viability and gene expression of the stem cell markers.

**Conclusion:**

Effect of hsa-miR-4270 inhibitor and mimic on the expression of the stem cell markers in GCSCs indicates that hsa-miR-4270 stimulates the stemness property of GCSCs, likely through stimulating the development of gastric stem cells.

## INTRODUCTION

Gastric cancer is one of the most common cancers with high mortality and low cure rate. Surgery and removal of tumor tissue, together with chemotherapy and radiotherapy, are the current treatments of GC^[^^1^^]^. However, the result of surgical resection is often unsuccessful, and the tumor cells spread to other tissues and distant organs of the body^[^^2^^]^. The uncontrolled proliferation of the cells and metastasis are the main causes of death in GC patients^[^^3^^]^. It has been well known that the unique and phenotypically distinct population of the cells, called CSCs or tumor-initiating cells, is found in malignant tumors. These cells have been demonstrated to be the primary reason for cancer cell growth, local invasion, and metastasis to distant tissues and are also responsible for disease resistance^[^^4^^]^. CSCs are a small heterogeneous population of cancer cells involved in tumor recurrence, growth, progression, differentiation, invasion, self-renewal of cancer^[^^5^^]^. These cells have the ability and characteristics similar to stem cells, including self-renewal and differentiation into various cell types^[^^6^^]^. The isolation and identification of CSCs in different types of cancers are highly important. In this regard, various methods have been developed including fluorescence-activated cell sorting and magnetic-activated cell sorting^[^^7^^]^. 

MicroRNAs or miRNAs are a subpopulation of short length noncoding RNAs involved in critical cell functions as post-transcriptional gene expression regulators^[^^8^^]^. There are a lot of documents indicating the important roles of miRNAs in the development of cancer stem cell, differentiation of cancer cells, and pathogenesis of cancer as oncogenes (oncomir) or tumor suppressor miRNAs^[^^9^^]^. Studies on the expression of miRNAs in CSCs and original cancer cells by microarray and RNA-seq analysis have demonstrated the significantly different expressions of miRNAs in CSCs and the parental cancer cells^[^^10^^,^^11^^]^. Using miRNA microarray analysis, investigations have shown that spheroid body-forming cells isolated from GC cell lines have a differently expressed miRNA profile from the parental cell lines^[^^11^^,^^12^^]^. One of the differentially expressed miRNAs in GCSCs is miR-4270, which is located in chromosome 3p25.1^[^^11^^]^. The miR-4270 is upregulated in CSCs and some carcinomas such as gastric, breast, and non-small-cell lung cancers^[^^13^^,^^14^^]^. However, a previous study has suggested that the downregulation of miR-4270 increases the cell proliferation, colony formation, and cell migration in lung adenocarcinoma^[^^15^^]^. 

CD44 is a transmembrane glycoprotein that interacts with hyaluronic acid of the extracellular matrix and pathologically associated with invasion and metastasis signaling pathways in various cancers, including GC^[^^16^^]^. OCT3/4, SOX2, Nanog, and KLF4 are CSC transcription factors that are involved in carcinogenesis and have been used to detect cancer stem cell subpopulations in various types of cancer^[^^17^^]^. In this study, we focused on the effect of miR-4270 inhibitor and mimic on the expression of stem cell marker genes, including *CD44, OCT3/4, SOX2,* *Nanog*, and *KLF4* in GCSCs derived from MKN-45 GC cell line. 

## MATERIALS AND METHODS

**GC cell culture**

The GC cell line (MKN-45) was purchased from the National Cell Bank of Iran (NCBI) affiliated to the Pasteur Institute of Iran, Tehran. The MKN-45 cell line was cultured in DMEM/F12 medium (Sigma-Aldrich) supplemented with 10% heat-inactivated FBS (Biochrom AG, Geramany) in the presence of 100 U/mL of penicillin and 100 μg/mL of streptomycin (both from Sigma-Aldrich). Cell cultures were incubated in a humidified incubator containing 95% air and 5% CO_2_ at 37 °C to reach ~80% confluence. The cell culture media were changed twice a week.

**GCSC isolation**

The surface of a 100-mm^2 ^cell culture dish was coated with a thin layer of agarose, and then MKN-45 cells (4 × 10^4^) were distributed in a suspension of single MKN-45 cells, which were cultured on the non-adhesive surface of the agarose-coated cell culture dish for two weeks. The culture media were replaced with fresh media three times a week. GCSCs were proliferated as floating colonies from the parental MKN-45 cell line^[^^18^^]^.

**Differentiation** **assays**

GCSCs derived from the MKN-45 cell line were cultured in a 60-mm^2 ^cell culture dish (5 × 10^3^ cells) in the DMEM/F12 medium supplemented with 10% FBS, 1 µM of dexamethasone, and 10 µg/mL of insulin as adipogenesis-inducing agents. The GCSC-derived cells were also treated with 100 nM of staurosporine as a neural-inducing agent. The GCSCs were induced for two weeks, and the culture media were refreshed three times a week. After two weeks, the colonies induced with adipogenic agents were stained with 0.6% (w/v) Oil Red O in 60% isopropanol at room temperature for 1 h, and then lipid vacuoles were observed under a microscope (AX-71, Olympus Corporation, Shinjuku-ku, Japan). Neural differentiation of GCSCs that had been treated with neural-inducing agents was observed under the same microscope after 24 h^[^^19^^,^^20^^]^. 

**hsa-** **miR-4270 inhibitor and mimic** **transfection**

Colonies of GCSCs were distributed as single cells on the non-adhesive surface of 96-well culture plates and then cultured at 5 × 10^3^ cells per well in an antibiotic-free DMED-F12 medium supplemented with 5% FBS and further incubated at 95% air and 5% CO_2_ at 37 °C for 24 h. GCSCs in the confluency of 50-60% were transfected with synthetic small, double-strandedRNA molecules, as well as hsa-miR-4270 inhibitor and mimic (Sigma-Aldrich), at several concentrations (0, 15, 20, 25, 30, 40, 50 and 60 nM) by Lipofectamine 2000 (Invitrogen, USA) according to the producer’s protocol for 24 h. 

**Cell viability assay**

Viability of the treated GCSCs with different concentrations of hsa-miR-4270 inhibitor and mimic was assessed by Trypan blue staining (Sigma-Aldrich). In summary, single GCSCs (5 × 10^3^) derived from MKN-45 cell line were plated on the non-adhesive surface of six-well plates containing 2 mL of DMEM/F12 supplemented with 10% FBS and different concentrations of hsa-miR-4270 inhibitor and mimic (0, 15, 20, 25, 30, 40, 50, and 60 nM) by Lipofectamine 2000 (Invitrogen) for 24 h. The next day, the GCSCs transfected with hsa-miR-4270 inhibitor and mimic were stained with Trypan blue, and viable cells (white) and non-viable cells (blue) were counted using a hemocytometer, consisting of nine 1 × 1 mm (1 mm^2^) squares, and a microscope (AX-71, Olympus Corporation, Shinjuku-ku, Japan). All experiments were conducted in triplicate and repeated three times.

**Quantitative real-time RT-PCR**

Total RNA extraction of the transfected GCSCs with 25 nM of hsa-miR-4270 inhibitor and mimic, along with the RNA of the untreated GCSCs, was performed by using RNeasy Plus Mini kit (Qiagen, USA) based on the manufacturer’s protocol. Synthesis of complementary DNA was carried out with the PrimeScript™ RT reagent Kit (Takara Bio Inc., Japan) according to the protocol recommended by the manufacturer. The complementary DNA synthesis of miRNA was performed by stem-loop primer as described before^[^^21^^]^. Primers of genes were acquired from our previous study (Table 1)^[^^22^^]^. A standard curve was drawn by two-fold serial dilution series to calculate the amplification efficiency of each primer. Quantitative gene expression was conducted by SYBR Premix Ex Taq II (Takara Bio Inc.) and *GAPDH* as an endogenous control gene in a Rotor-Gene 3000 System (Corbett Research, Australia). Quantitave real-time RT-PCR data analysis was conducted in accordance with a formerly described method^[^^23^^]^.

**Statistical** **analysis**

The results of all quantitative experiments were evaluated by student’s t-test and one-way ANOVA. Data were displayed as the mean ± SEM. p value <0.05 was considered as statistically significant.

**Table 1:** Primer sequences used in real-time RT-PCR

**Products length (bp)**	**Ta** **(°C)**	**Primers length (bp)**	**Sequences ** ** 5'** **3ˊ**	**Primers** **name**	**Genes**
162	54	25	ACTCTGGTAAAGTGGATATTGTTGC	Sence	*GAPDH*
		21	GGAAGATGGTGATGGGATTTC	Antisence	
					
169	54	18	GCTTCAGGGTTTCATCCA	Sence	*Oct3/4*
		18	GGCGGCAATCATCCTCTG	Antisence	
					
129	53	20	TAACAGTTCCTGCATGGGCGGC	Sence	*CD44*
		21	CGTGCAAATTCACCAGAAGGC	Antisence	
					
115	54	24	CAACATCACAGAGGAAGTAGACTG	Sence	*Sox2*
		19	CCTTGGCATGAGATGCAGG	Antisence	
					
167	59	21	AACTCTCCAACATCCTGAACC	Sence	*Nanog*
		22	GTGGTAGGAAGAGTAAAGGCTG	Antisence	
					
171	59	21	GAACCCACACAGGTGAGAAAC	Sence	*KLF4*
		19	TGTGTAAGGCGAGGTGGTC	Antisence	
					
70	58	18	TCAGGGAGTCAGGGGAGG	Sence	*miR*-*4270*
		19	GAACATGTCTGCGTATCTC	Antisence	
		50	GTCGTATCCAGTGCAGGGTCCGAGGTATTCGCACTGGATACGACGCCCTC	RT	

Ta, annealing temperature; RT, reverse transcription

**Fig. 1:**
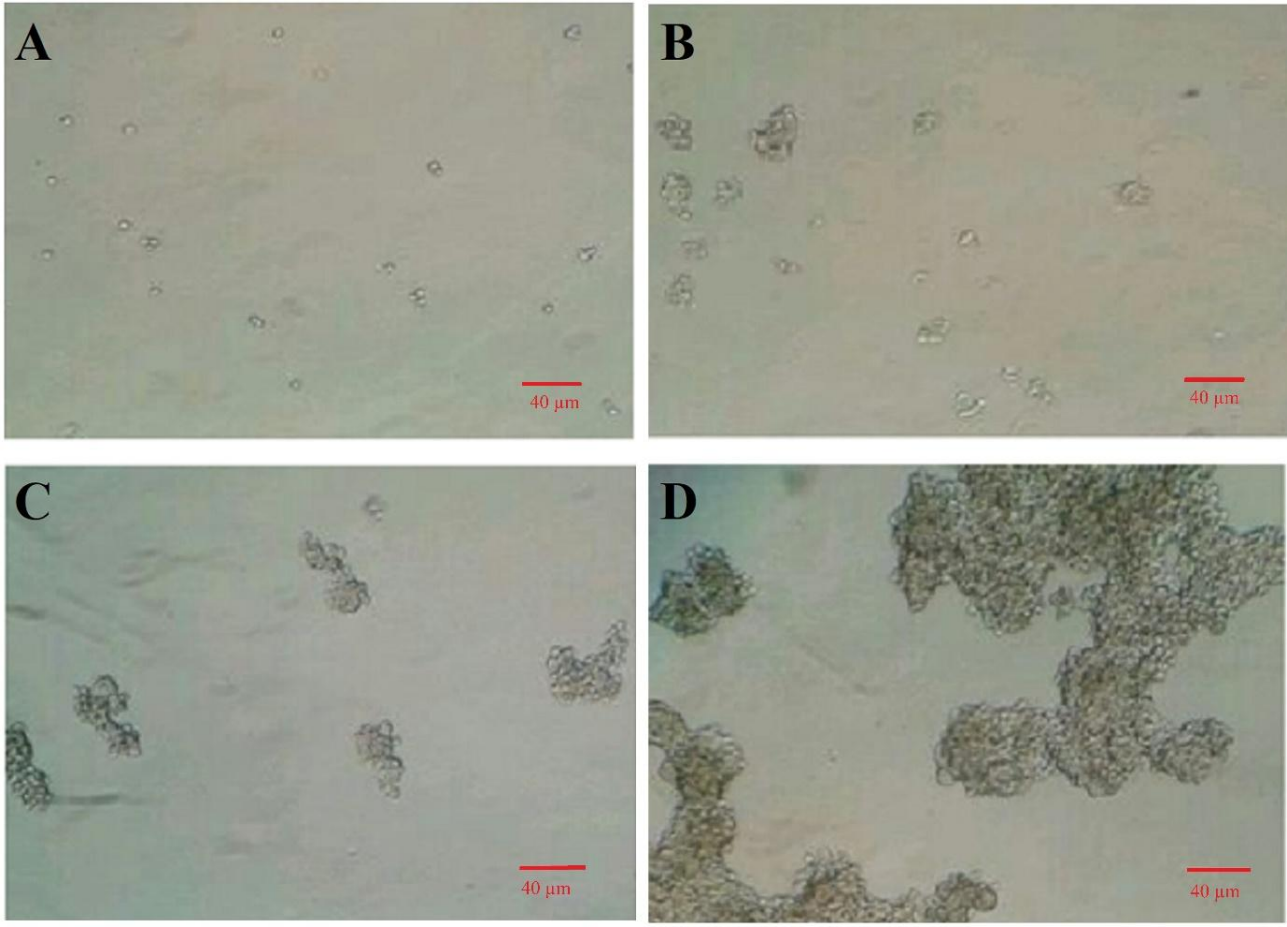
Isolation of GCSCs from the MNK-45 cell line. (A) A suspension of single cells of the MKN-45 cell line cultured on a thin layer of agarose; (B) spheroid cancer cells proliferated and produced small flowing colonies after four days; (C) small flowing colonies grown after seven days; (D) colonies became larger and larger in two weeks (magnification 100×)

## RESULTS

**Isolation of GCSCs from MKN-45 cell line**

Isolation of GCSCs from MNK-45 cell line was performed according to our previous study^[^^18^^]^. Briefly, a suspension of MKN-45 cells was cultured on a non-adhesive surface of culture plate for about two weeks to form GCSC colonies. As indicated in Figure 1A, a suspension of the single cells of MKN-45 was cultured on a thin layer of agarose, and spheroid cancer cells with stemness properties were proliferated and produced in small colonies at day four (Fig. 1B). These colonies were grown in one week (Fig. 1C) and became larger and larger in two weeks (Fig. 1D). 

**In vitro differentiation of GCSCs**

**Fig. 2:**
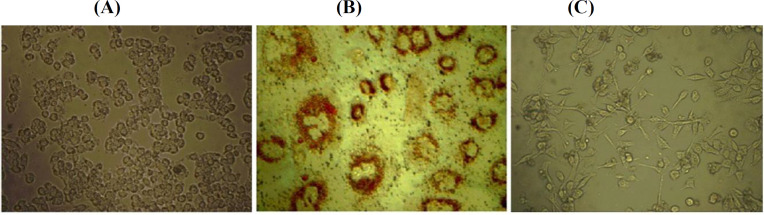
Differentiation of GCSCs. GCSCs derived from MKN-45 cell line were cultured at 5×10^3^ cells in a 60-mm^2 ^cell culture dish in DMEM/F12 supplemented with 10% FBS and 1 µM of dexamethasone and 10 µg/mL of insulin as adipogenesis-inducing agents and 100 nM of staurosporine as a neural-inducing agent. (A) Untreated GCSCs; (B) Oil Red O staining of GCSCs treated with dexamethasone and insulin for two weeks, and lipid vacuoles in adipose cells were detected under a microscope (AX-71, Olympus, Japan); (C) GCSCs treated with staurosporine for 24 h, and neural cells were detected under the same microscope (magnification 200×).

To support the GCSCs properties and evaluate the differentiation ability of cancer stem cells, we utilized in vitro differentiation assay. GCSCs derived from MKN-45 cells were differentiated into adipose cells after two weeks of induction by adipogenesis-inducing agents. Untreated GCSCs are shown in Figure 2A. Lipid vacuoles in adipose cells were detected by staining with Oil Red O (Fig. 2B). The GCSCs were also differentiated into neural cells within 24 h by exposure to staurosporine (Fig. 2C).

**E** **ffect** **of hsa-miR-4270 on** **GCSC cell viability**

**Fig. 3:**
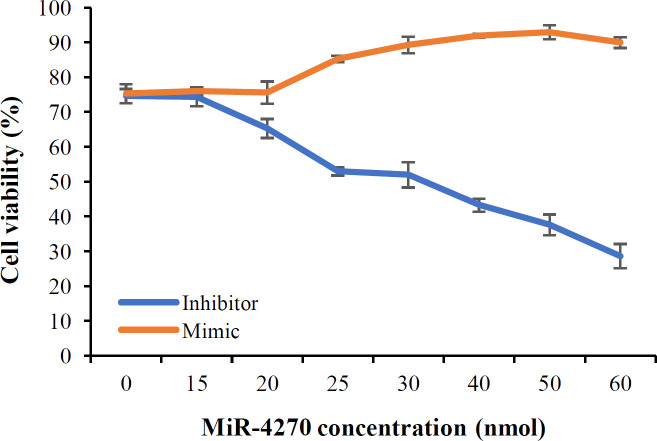
Cell viability assay of GCSCs by Trypan blue staining. MiR-4270 inhibitor reduced the cell viability of GCSCs. In opposite, the cell viability of GCSCs was increased by treating with hsa-miR-4270 mimic (p < 0.05 vs. control group, ANOVA analysis)

To investigate the effect of miR-4270 inhibitor and mimic on the cell viability of cancer stem cells, GCSCs were transfected with different concentrations of miR-4270 inhibitor and mimic. Cell viability of the treated GCSCs with hsa-miR-4270 inhibitor and mimic were measured by Trypan blue staining. GCSCs were treated with hsa-miR-4270 inhibitor and mimic at the concentrations of 0, 15, 20, 25, 30, 40, 50, and 60 nM for 24 h. Staining of GCSCs by Trypan blue showed that treatment of GCSCs with hsa-miR-4270 inhibitor declined the cell viability in a concentration-dependent manner with the IC_50_ value of 30 pmol, while treating the GCSC with hsa-miR-4270 mimic increased the cell viability (Fig. 3). Moreover, it was found that hsa-miR-4270 mimic could neutralize the effect of hsa-miR-4270 inhibitor. In the gene expression experiment, we used 25 pmol of hsa-miR-4270 inhibitor and mimic, which had the minimum cytotoxicity on the cells and significant concentration differences between treated and untreated cells. 

**Effect of hsa-miR-** **4270** **on** **stemness gene expression**

**Fig. 4:**
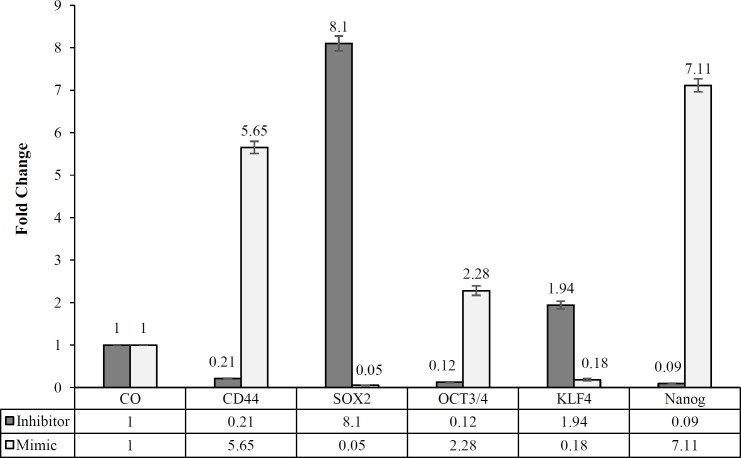
Evaluation the effect of hsa-miR-4270 inhibition and mimic on the gene expression of stem cell marker genes and *GAPDH* as the internal control by real-time RT-PCR (^*^p < 0.05 vs. control group, Student’s t-test analysis)

To study the effect of hsa-miR-4270 inhibitor and mimic on the stemness feature of GCSCs, we analyzed the gene expression of some stemness markers, including *CD44*, *OCT3/4*, *SOX2*, *Nanog*, and *KLF4 *by using real-time RT-PCR. The gene expression analysis of the GCSCs, treated with 25 pmol of hsa-miR-4270 inhibitor for 24 h, showed 86%, 79%, and 91% decreases in the expression of *OCT3/4*, *CD44*, and *Nanog*, respectively. In contrast, the transcription levels of *SOX2* and *KLF4* were respectively 8.1 and 1.94 folds higher in the treated GCSCs with 25 pmols of hsa-miR-4270 inhibitor than the untreated GCSCs (Fig. 4). The gene expression analysis of the GCSCs treated with 25 pmol of hsa-miR-4270 mimic exhibited opposite results, as we expected. The expression of *OCT3/4*, *CD44*, and *Nanog* respectively increased 2.28, 5.65, and 7.11 folds in the treated GCSCs, while that of *SOX2* and *KLF4* showed 95% and 82% reductions in the treated GCSCs, respectively (Fig. 4).

## DISCUSSION

 Cancer stem cells, a small subpopulation of tumor cells with self-renewal ability and differentiation, comprise the most essential characteristics related to tumor growth and metastasis^[^^24^^,^^25^^]^. Hence, one approach to control cancer growth and development is to reduce the stemness properties of cancer cells^[^^26^^]^. 

There are many investigations showing that noncoding RNAs, such as circular RNA and miRNAs, inhibit the tumorigenicity and metastasis in a variety of cancers, including gastric, breast, ovarian, lung, and skin carcinomas^[^^27^^]^. Peng and coworkers^[^^28^^]^ have indicated that miR-191 and miR-425 expression levels are correlated with tumor stage and metastatic state of GC. Huang et al.^[^^29^^]^ have explored that miR-373 and miR-520c can stimulate tumor invasion and metastasis in breast cancer cell lines. Based on our knowledge, there is scant study on the effect of miR-4270 on various cancers, and only one study has indicated the upregulation of miR-4270 in GCSCs^[^^11^^]^. In this study, we aimed to investigate the effect of hsa-miR-4270 inhibitor and mimic on the expression of stem cell marker genes, including *CD44, OCT3/4, SOX2, KLF4*, and *Nanog* in GCSCs derived from MKN-45 cell line. Therefore, we treated GCSCs with several concentrations of hsa-miR-4270 inhibitor and mimic and found that 25 pmol concentration had the minimum cytotoxicity on the cells and significant concentration differences between treated and untreated cells. Considering this outcome, we treated GCSCs with 25 pmol of hsa-miR-4270 inhibitor and mimic. We also used an inexpensive non-adhesive cell culture without using flow cytometry or growth factors to isolate GCSCs from MKN-45 cell line^[^^7^^,^^30^^]^. The differentiation ability of CSCs has been investigated in various studies. Xiaomeng Xu and colleagues^[^^20^^]^ have suggested that CSCs isolated from the tissue of patients differentiate into fat and neural cells. Zengfu Xue et al.^[^^19^^]^ have also differentiated CSCs that were isolated from gastric cancer cells (SGC7901) into endothelial cells. In our study, GCSCs derived from MKN-45 cell line under induced conditions, including dexamethasone and insulin as adipogenesis-inducing agents and staurosporine as neural-inducing agent, were differentiated into fat and neural cells. MiRNAs can affect some genes indirectly or by a mediator to change the cell fate to being cancerous or normal^[^^31^^]^. Tay and co-workers^[^^32^^]^ have shown that three miRNAs, including miR-134, miR-296, and miR-470, could impede the expression of *NANOG*,* SOX2*, and *OCT3/4* in mouse embryonic stem cells. In this study, we investigated the effect of hsa-miR-4270 on stemness features by evaluating the expression level of stem cell markers such as *CD44*, *OCT3/4*, *SOX2*, *KLF4*, and *Nanog* under the treatment of hsa-miR-4270 inhibitor and mimic. Similar to our study, other studies examined the expression of stem cell marker genes to show changes in the stemness features of CSCs in breast and colorectal cancers, as well as GC. Otsubo et al.^[^^33^^]^ have indicated that miR-126 could suppress *SOX2 *expression and contribute to stomach carcinogenesis. Huang and colleagues^[^^29^^]^ have found that miR-520c and miR-373 suppress CD44 and induce in vitro and in vivo cell invasion and migration in breast tumor cell line. 

The quantitative RT-PCR data analysis displayed that upon treatment with hsa-miR-4270 inhibitor, the expression of *CD44*, *OCT3/4*, and *Nanog* genes reduced, while that of *SOX2* and *KLF4* genes increased, but following treating with miR-4270 mimic, this trend was reverse. According to these results, hsa-miR-4270 inhibition can reduce the stemness features of stem cells, which in turn diminishes the cell proliferation. We also found opposite results in the treatment of GCSCs with hsa-miR-4270 mimic, which increased the stemness feature of GCSCs. 

The study of the effect of hsa-miR-4270 inhibitor and mimic on the expression of stem cell markers such as *CD44*, *OCT3/4*, *SOX2*, *KLF4*, and *Nanog* in GCSCs indicated that hsa-miR-4270 stimulated the stemness property of GCSCs. Our findings show that hsa-miR-4270 inhibitor decreases the stemness feature of GCSCs and represses the expression of hsa-miR-4270, which would be helpful in the treatment of GC. In our opinion, miR-4270 inhibitor declined the expression of miR-4270; therefore, it could be used in the treatment of GC patients in future. For validation of our investigation, it is necessary to study the cell signaling of miR-4270 and also evaluate the effect of miR-4270 inhibitor and mimic on animals. 

## DECLARATIONS

### Acknowledgments

The financial support of this by the Vice-Chancellor of the Research Department of Shiraz University of Medical Sciences (Shiraz, Iran) is acknowledged. The authors also would like to thank Fatemeh Mahmoodi for her contribution to complete this work. 

### Ethical statement

All authors have read and approved the contents of the final manuscript and agreed to publicate this manuscript. 

### Data availability

The data supporting the findings of this study are available on request from the corresponding author. The data are not publicly available due to privacy or ethical restrictions.

### Author contributions

HA: designed, directed and conducted the investigation and wrote the manuscript; SAS: contributed to cell culture; YN: contributed to quantitative real-time RT-PCR; MF: contributed to writing and revising the manuscript. 

### Conflicts of interest:

None declared.

### Funding/support

This work was supported by the Vice-Chancellor of the Research Department of Shiraz University of Medical Sciences, Shiraz, Iran (no. 24466).

## References

[1] Sitarz R, Skierucha M, Mielko J, Offerhaus GJA, Maciejewski R, Polkowski WP (2018). Gastric cancer: epidemiology, prevention, classifcation, and treatment. Cancer management and research.

[2] Uchihara T, Ishimoto T, Yonemura A, Baba H (2018). Therapeutic targets against gastric cancer stem cells interacting with tumor microenvironment. Journal of cancer metastasis and treatment.

[3] Nagini S (2012). Carcinoma of the stomach: A review of epidemiology, pathogenesis, molecular genetics and chemoprevention. World journal of gastrointestinal oncology.

[4] Michael Baumann MK, Richard Hill (2008). Exploring the role of cancer stem cells in radioresistance. Nature review.

[5] Aponte PM, Caicedo A (2017). Stemness in cancer: Stem cells, cancer stem cells, and their microenvironment. Stem cells international.

[6] Tannishtha Reya SJM, Michael F (2001). Clarke, Irving L. Weissman. Stem cells, cancer, and cancer stem cells. Nature.

[7] Liu J, Ma L, Xu J, Liu C, Zhang J, Liu J, Chen R, Zhou Y (2013). Spheroid body-forming cells in the human gastric cancer cell line MKN-45 possess cancer stem cell properties. Stem cells international.

[8] Paolis VD, Lorefice E, Orecchini E, Carissimi C, Laudadio I, Fulci V (2021). Epitranscriptomics: A New layer of microRNA regulation in cancer. Cancers.

[9] Garg M (2012). MicroRNAs, stem cells and cancer stem cells. World journal of stem cells.

[10] Zhu; H-R, Huang; R-Z, Yu; X-N, Shi; X, Bilegsaikhan; E, Guo; H-Y, Song GQ, Weng SQ, Dong L, Janssen HLA, Shen XZ, Zhu JM (2018). Microarray Expression Profling of microRNAs Reveals Potential Biomarkers for Hepatocellular Carcinoma. Tohoku journal of experimental medicine.

[11] Liu J, Ma L, Wang Z, Wang L, Liu C, Chen R, Zhang J (2014). MicroRNA expression profile of gastric cancer stem cells in the MKN-45 cancer cell line. Acta biochimica et biophysica Sinica.

[12] Golestaneh AF, Atashi A, Langroudi L, Shafiee A, Ghaemi N, Soleimani M (2012). miRNAs expressed differently in cancer stem cells and cancer cells of human gastric cancer cell line MKN-45. Cell biochemistry and function.

[13] Wang H, Huang Z, Zhao X, Guo B, Ji Z (2020). miR-4270 regulates cell proliferation and apoptosis in patients with Sertoli cell-only syndrome by targeting GADD45A and inactivating the NOTCH signaling pathway. American journal of translational research.

[14] Shen D, Zhao H, Zeng P, Song J, Yang Y, Gu X (2020). Circular RNA has-circ-0005556 accelerates gastric cancer progression by sponging miR-4270 to Increase MMP19 expression. Joural of gastric cancer.

[15] Sun G, Ding X, Bi N, Wang Z, Wu L, Zhou W, Zhao Z, Wang J, Zhang W, Fan J, Zhang W, Dong X, Lv N, Song Y, Zhan Q, Wang L (2019). Molecular predictors of brain metastasisrelated microRNAs in lung adenocarcinoma. PLoS genetics.

[16] Jang BI, Li Y, Graham DY, Cen P (2011). The role of CD44 in the pathogenesis, diagnosis, and therapy of gastric cancer. Gut and liver.

[17] van Schaijik B, Davis PF, Wickremesekera AC, Tan ST, Itinteang T (2018). Subcellular localisation of the stem cell markers OCT4, SOX2, NANOG, KLF4 and c-MYC in cancer: a review. Journal of clinical pathology.

[18] Fatemeh Mahmoodi, Akrami H (2017). PlGF Knockdown decreases tumorigenicity and stemness properties of spheroid body cells derived from gastric cancer cells. Journal of cellular biochemistry.

[19] Zengfu Xue HY, Juntang Li, Shuli Liang, Xiqiang Cai, Xiong Chen, Qiong Wu, Liucun Gao, Kaichun Wu, Yongzhan Nie, Daiming Fan (2012). Identification of cancer stem cells in vincristine preconditioned SGC7901 gastric cancer cell line. Journal of cellular biochemistry.

[20] Xiaomeng Xu XZ, Sheng Wang, Hui Qian, Wei Zhu, Huiling Cao, Mei Wang, Yuan Chen, Wenrong Xu (2011). Isolation and comparison of mesenchymal stem-like cells from human gastric cancer and adjacent non-cancerous tissues. Journal of cancer research and clinical oncology.

[21] Chen C, Ridzon DA, Broomer AJ, Zhou Z, Lee DH, Nguyen JT, Barbisin M, Lan Xu N, Mahuvakar VR, Andersen MR, Lao KQ, Livak KJ, Guegler KJ (2005). Real-time quantification of microRNAs by stem–loop RT–PCR. Nucleic acids research.

[22] Akrami H, Moradi B, Borzabadi Farahani D, Mehdizadeh K (2018). Ibuprofen reduces cell proliferation through inhibiting Wnt/β catenin signaling pathway in gastric cancer stem cells. Cell biology international.

[23] Livak KJ, Schmittgen TD (2001). Analysis of relative gene expression data using realtime quantitative PCR and the 2-DDCT method. Methods.

[24] Peitzsch C, Tyutyunnykova A, Pantel K, Dubrovska A (2017). Cancer stem cells: The root of tumor recurrence and metastases. Seminars in cancer biology.

[25] Xia P, Xu XY (2017). Epithelial-mesenchymal transition and gastric cancer stem cell. Tumour biology.

[26] Fu L, Bu L, Yasuda T, Koiwa M, Akiyama T, Uchihara T, Baba H, Ishimoto T (2020). Gastric cancer stem cells: current insights into the immune microenvironment and therapeutic targets. Biomedicines.

[27] Huiwen Yan, Bu P (2018). Non-coding RNAs in cancer stem cells. Cancer letters.

[28] Peng W Z, Ma R, Wang F, Yu J, Liu Z B (2014). Role of miR-191/425 cluster in tumorigenesis and diagnosis of Gastric Cancer. International journal of molecular sciences.

[29] Huang Q, Gumireddy K, Schrier M, Sage Cl, Nagel R, Nair S, Egan DA, Li A, Huang G, Klein Szanto AJ, Gimotty PA, Katsaros D, Coukos G, Zhang L, PuréE, Agami R (2008). The microRNAs miR-373 and miR-520c promote tumour invasion and metastasis. Nature cell biology.

[30] Mahmoodi F, Akrami H (2017). PlGF Knockdown decreases tumorigenicity and stemness properties of spheroid body cells derived from gastric cancer cells. Journal of cellular biochemistry.

[31] Zhang Z, Li Z, Li Y, Zang A (2014). MicroRNA and signaling pathways in gastric cancer. Cancer gene therapy.

[32] Tay Y, Zhang J, Thomson AM, Lim B, Rigoutsos I (2008). MicroRNAs to Nanog, Oct4 and Sox2 coding regions modulate embryonic stem cell differentiation. Nature.

[33] Otsubo T, Akiyama Y, Hashimoto Y, Shimada S, Goto K, Yuasa Y (2011). MicroRNA-126 inhibits SOX2 expression and contributes to gastric carcinogenesis. PloS one.

